# *Polygonati Rhizoma* Polysaccharides Ameliorated Diabetic Kidney Disease in *db/db* Mice via Inhibiting TGF*β* /Smad2 Signaling Pathway

**DOI:** 10.1002/fsn3.70677

**Published:** 2025-07-18

**Authors:** Xiyu Mei, Zeming Ren, Ziyun Gao, Sisi Chen, Xuan Chen, Qingyun Zhou, Yeling Tong, Guanhai Dai

**Affiliations:** ^1^ Institute of Basic Medicine, Zhejiang Academy of Traditional Chinese Medicine Hangzhou China; ^2^ The Key Laboratory of Pharmacodynamic Material Basis Research in Chinese Medicine of Zhejiang Province Tongde Hospital of Zhejiang Province Hangzhou China; ^3^ School of Basic Medical Sciences and Forensic Medicine Hangzhou Medical College Zhejiang Hangzhou China; ^4^ Second Clinical Medical College, Zhejiang Chinese Medical University Hangzhou China

**Keywords:** *db/db* mice, diabetic kidney disease, *Polygonati Rhizoma* polysaccharides, Smad7, TGF*β*/Smad2

## Abstract

*Polygonati Rhizoma* polysaccharides (PP) are the active components isolated from medicine food homology (MFH) Chinese herb *Polygonati Rhizoma*. Diabetic kidney disease (DKD) is a serious diabetic complication occurring in the later stage of diabetes. We have previously reported the hypoglycemic effect of PP on early‐stage type 2 diabetes (T2D). This study evaluated the effect of PP on DKD and investigated the underlying mechanisms. PP was mainly composed of L‐fucose, L‐rhamnose, D‐glucose, and D‐mannose. Ten‐week‐old Leprdb/Leprdb (*db/db*) compared with control Leprdb/Lepr+ (*db*/*+*) mice were given vehicle or PP for 16 weeks. Our results showed that PP alleviated renal dysfunction (BUN, Cr, microalbumin) and pathological damage in *db/db* mice. PP improved lipid homeostasis (TG, NEFA, and HDL) and reversed oxidative stress (CAT, MDA, SOD, and GSH) in the serum of *db/db* mice. Besides, PP reduced the Masson‐positive area and expression of TGF*β* in the kidneys from *db/db* mice. Furthermore, PP upregulated the expression of Smad7 and abrogated the phosphorylation of Smad2 in the kidneys from *db/db* mice. In conclusion, PP ameliorated kidney dysfunction and fibrosis through attenuating dyslipidemia, anti‐oxidative stress, and inhibiting the activation of TGF*β*/Smad2 signaling pathway in *db/db* mice. The study provides a theoretical basis for further elucidation of the effect and mechanism of PP in attenuating DKD.

## Graphical abstract

PP ameliorated diabetic kidney disease in T2D *db/db* mice. PP improved the lipid metabolism and attenuated the oxidative stress in T2D *db/db* mice. PP increased the expression of Smad7 and inhibited the activation of TGF*β*/Smad2 signaling pathway in kidneys from T2D *db/db* mice.

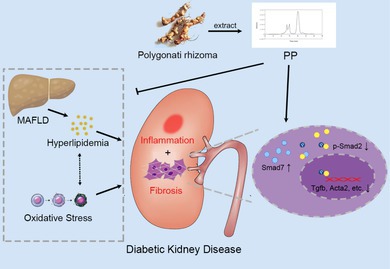

## 1 Introduction

As the International Diabetes Federation estimated, 784 million people will live with diabetes by 2045, while type 2 diabetes (T2D) accounts for over 90% of all diabetes cases (Ahmad et al. 2022). Most patients with T2D suffer at least one diabetic complication, such as dysfunctions of the kidney, retina, cardiovascular system, neurons, and liver (Zheng et al. 2018). These complications are the leading cause of mortality in patients with T2D, and there is a lack of effective therapy that can reverse this injury (Demir et al. 2021). Diabetic kidney disease (DKD) is one of the most prevalent comorbidities of diabetes and the leading cause (44.5%) of end‐stage renal disease (ESRD) (KDIGO Diabetes Work Group 2022; Thomas et al. 2015). Improved glycemic control has led to the major decline in the incidence of DKD over the past 30 years, but patients with diabetes still develop DKD as diabetes mellitus (DM) progresses (Thomas et al. 2015). At least half of patients with T2D will develop DKD over the course of their lifetime and further advance to kidney failure requiring dialysis (KDIGO 2022; Shigidi and Karrar 2021).

DKD is a complex disease process that involves several pathophysiologic mechanisms such as insulin resistance, chronic inflammation, and oxidative stress, eventually leading to kidney damage and fibrosis (Jung and Yoo 2022). In the early stage of DKD, the majority of patients develop intraglomerular hypertension and single‐nephron hyperfiltration before exhibiting kidney function decline (Lytvyn et al. 2020). Previous studies showed the angiotensin‐converting enzyme inhibitor (ACEI) treatment might have benefits on reducing albuminuria, as well as adverse effects like dry cough and acute kidney injury (Anders et al. 2018). However, there remains an unmet need for treatment to prevent and arrest DKD. In order to ameliorate DKD and avoid the side effects of drugs, people facing progressive DKD and kidney failure in particular may be highly motivated to implement nutrition solutions (KDIGO Diabetes Work Group 2022).

*Polygonati Rhizoma* is the dried rhizome of *Polygonatum sibiricum* Delar. ex Redoute, *Polygonatum cyrtonema* Hua, or *Polygonatum kingianum* Coll. et Hemsl. (Chinese Pharmacopeia Commission 2020). *Polygonati Rhizoma* has been widely used as a medicine food homology (MFH) in China for thousands of years. In Traditional Chinese Medicine (TCM), *Polygonati Rhizoma* is commonly used to ameliorate metabolic problems including obesity and T2D (Zhao et al. 2018). Previous studies suggest *Polygonati Rhizoma* has immunomodulatory, anti‐diabetic, neuroprotective, and anti‐fatigue effects (Gong et al. 2023). *Polygonati Rhizoma* polysaccharides (PP) are the major active component in *Polygonati Rhizoma* indexed in the China Pharmacopeia (Chinese Pharmacopeia Commission 2020; Gong et al. 2023). Previous studies showed PP had a variety of biological benefits including anti‐aging, anti‐diabetes, anti‐inflammatory, anti‐osteoporosis, anti‐atherosclerotic, and neuronal protection (Gong et al. 2023; Shen et al. 2021). We have reported that PP ameliorated hyperglycemia in a batch of 15‐week‐old diabetic *db/db* mice (Chen et al. 2023). However, the effect of PP on DKD was still unknown. In this study, the effect of PP on DKD was observed, and the involved mechanisms were investigated.

## 2 Materials and Methods

### 2.1 Preparation of PP

PP (polysaccharide content > 70%) was purchased from Shanghai Yuanye Bio‐Technology Co. Ltd. (Shanghai, China). In brief, *Polygonati Rhizoma* (dried rhizome of *Polygonatum cyrtonema* Hua) was purchased from Huadong Medicine Group Co. Ltd. (Hangzhou, China). The powder of *Polygonati Rhizoma* was pulverized and defatted, then extracted under reflux by water for 2 h three times. Ethanol was added to the solution to 75%, and the supernatant was collected. Adding ethanol to the supernatant to a final concentration of 90%, the precipitate was obtained by centrifugation. Finally, the precipitate was treated with 10% TCA (w/v) to remove protein and lyophilized. The total polysaccharide content was measured by the phenol–sulfuric acid colorimetric method as previously described (Dubois et al. 1951). Glucose was used as the reference standard for polysaccharide measurement. PP powder was stored at −20°C. We prepared PP solution by using distilled deionized water (ddH_2_O) every day.

### 2.2 Molecular‐Weight Distribution and Monosaccharide Profile

A high‐performance liquid chromatography instrument (HPLC; Shimadzu) equipped with a differential detector (RID‐20) was adopted in high‐performance gel permeation chromatography (HPGPC) analysis to determine the molecular weight of PP. The sample was separated on a connection of three gel columns (TSKgel G3000PWXL, TSKgel G4000PWXL, and TSKgel G5000PWXL, 7.8 × 300 mm). The concentration of the configured sample was 2 mg/mL. The sample was completely dissolved in deionized water before filtering by using a 0.22 μM aqueous membrane. The flow rate was 0.6 mL/min, and the column temperature was set at 35°C. Dextran standards with molecular weights of 3620, 7000, 9000, 12,600, 20,000, 50,000, 126,000, 250,000, 490,000, and 1,000,000 Da were used to calibrate the chromatographic column and establish a standard curve. The standard curve is the molecular weight of the elution volume of standard glucan on HPGPC. Signals from 190 nm to 400 nm were observed, and the spectra (335 nm) were recorded.

For the monosaccharide profile, PP samples were hydrolyzed with trifluoroacetic acid (2.0 M), and the hydrolysate was then concentrated under reduced pressure with methanol to remove trifluoroacetic acid. Derivatization was performed by using NaOH and 1‐phenyl‐3‐methyl‐5‐pyrazolone (PMP) in methanol. HPLC analysis was then performed (0.25 mL/min, 30°C) and the chromatograms of the standards are shown in Figure S1.

### 2.3 Experimental Animals

Fourteen male C57BL/6^*db/db*^ mice (10‐weeks old) and seven male C57BL/6^*db/*/+^ mice (10‐weeks old) were purchased from Changzhou Cavens Experimental Animal Co. Ltd. (Changzhou, China). Since type 2 diabetes modeling in female mice is unstable, male mice were used in this study. Animals were housed under controlled temperature (23°C ± 2°C), humidity (50%), and lighting (12 h light/12 h dark). All animals received humane care according to the institutional animal care guidelines approved by the Laboratory Animal Welfare Ethics Committee of the Zhejiang Academy of Traditional Chinese Medicine (Approval No: 2022‐048).

### 2.4 Treatment of Animals

Fourteen *db/db* mice (blood glucose concentration > 16.5 mM/L) were randomly divided into the *db/db* model group (*n* = 7) and the *db/db* + PP group (*n* = 7), respectively. The *db/+* mice were assigned to the negative control (NC) group (*n* = 7). The yield of PP from *Polygonati Rhizoma* was 10.52%. Referring to previous studies (Wang et al. 2019; Li, Fang, et al. 2020; Kato and Miura 1993), the administration dose of PP was 1.0 g/kg. The *db/db* + PP group was treated with PP (1.0 g/kg, i.g.) consecutively for 16 weeks. An equal volume of vehicle (ddH_2_O) was given to control mice and *db/db* model mice. The body weight, the water and food uptake, and the level of blood glucose were recorded. Urine samples were collected at 12 weeks of PP treatment. After the animal experiment, whole blood was collected from the abdominal aorta of narcotized mice (sodium pentobarbital, 50 mg/kg, i.p), and then the mice were killed by cervical dislocation, and the kidneys were harvested. Experiments without narcotizing were performed by using gentle holding and kind soothing.

### 2.5 Extraction and Culture of Primary Glomerular Endothelial Cells (GECs)

After PP or solvent treatment, GECs were isolated from *db/db* and *db/+* mice as previously described (Chen et al. 2022). The GECs were cultured in ECM medium (ScienCell) supplemented with 5% [v/v] fetal bovine serum and 1% endothelial cell growth supplement (ECGS), 100 U/mL penicillin, and 100 μg/mL streptomycin.

### 2.6 Enzyme‐Linked Immunosorbent Assay (ELISA)

The supernatants from tissue homogenate were collected by centrifugation (3000 *g*, 4°C, 10 min). Concentration of insulin, leptin (LEP), and IL‐6 in tissues was then detected according to the instructions from the manufacturer (Proteintech, Wuhan, China). The optical density of each well at 450 nm was determined, and the results were corrected by using BCA Protein Assay.

### 2.7 Analysis of Serum Biochemical Indicators

Serum was collected by centrifugation (3000 rpm, 15 min, RT). Serum contents of glucosylated serum protein (GSP), triglyceride (TG), nonesterified fatty acids (NEFA), high density lipoprotein cholesterol (HDL‐C), blood urea nitrogen (BUN), creatinine (Cr), and alanine/aspartate aminotransferases (ALT/AST) activity, malondialdehyde (MDA), catalase (CAT), total superoxide dismutase (T‐SOD), and glutathion peroxidase (GSH‐Px) were measured according to the instructions from the manufacturer (Nanjing Jiancheng, Nanjing, China).

### 2.8 Reactive Oxygen Species (ROS) Amount Measurement

ROS amount was measured and calculated as previously described (Pang et al. 2016). Cells were lysed and centrifuged for 3 min (4°C, 1000 *g*) to collect the supernatant. The measurement was carried out at 520 nm with excitation at 488 nm.

### 2.9 BODIPY Fluorescence Staining

BODIPY fluorescence staining in mice hepatocyte AML‐12 cells was conducted as previously described (Lin et al. 2020). Cells were pre‐treated with 0.5 mM NEFA (oleic acid + palmitic acid = 0.5 mM, oleic acid: palmitic acid = 2:1) and PP (10, 30 μg/mL) for 24 h. Otherwise, cells were co‐incubated with H_2_O_2_ (100 μM) and/or PP (30 μg/mL) for 2 h before cells were treated with 0.5 mM NEFA for another 24 h.

### 2.10 Urine Biochemistry Measurement

Overnight urine samples were collected from each mouse in the metabolic cage at the 12th week after PP‐treat. The urine samples were centrifuged (4°C, 5 min, 1500 rpm) and then discarded the precipitate. Urinary microalbumin (mAlb), Cr, and total protein levels were measured according to manufacturers' instructions (Nanjing Jiancheng, Nanjing, China).

### 2.11 Histological Analysis

For renal histological analysis, kidneys were fixed with 4% paraformaldehyde. Two sections (4 μm) were cut from each sample. One section was stained with hematoxylin and eosin (H&E). The other section was subjected to Masson's staining. The glomerular injury was assessed by a semiquantitative analysis using the method of Raij et al. (Raij et al. 1984); at least 5 fields of view were assessed in an individual sample. For hepatic histological analysis, livers were fixed with 4% paraformaldehyde. Two sections (4 μm) were cut from each sample. One was stained with H&E, and the other was stained with periodic acid‐schiff (PAS).

### 2.12 Real‐Time PCR Assay

Total RNA was isolated from renal tissues by using Trizol reagents (Thermo Fisher Scientific, Waltham, MA). The RNA content was determined by measuring the optical density at 260 nm. Synthesis of cDNA and real‐time PCR was performed by using the PrimeScript RT Master Mix kit and SYBR Premix Ex Taq kit (Takara, Shiga, Japan). The relative expression of target genes was normalized to *β‐Actin*. The results were analyzed by the $2^{-\Delta \Delta C^{t}}$ method and given as a ratio compared with the control. The primer sequences were presented in Table S1.

### 2.13 Protein Extraction and Capillary Immunoassays

Renal proteins were homogenized and isolated in ice‐cold lysis buffer as previously described (Chen et al. 2022). Protein concentrations were determined using the BCA Protein Assay Kit (Thermo Fisher Scientific, Waltham, MA). A partially automated Simple Western system was used to analyze the protein expression of renal tissues based on capillary electrophoresis (Nguyen et al. 2011). 12–230 kDa separation capillary cartridges, the anti‐rabbit detection module, and the anti‐mouse detection module were from Protein Simple (San Jose, CA). Antibodies for glyceraldehyde‐3‐phosphate dehydrogenase (GAPDH, internal control) and transforming growth factor‐*β* (TGF*β*) were from Proteintech (Wuhan, China). Antibodies for tumor necrosis factor (TNF*α*) and Smad7 were from Affinity (Liyang, China). Antibodies for Smad2 and phospholated‐Smad2 (Ser465/467) were from Cell Signaling Technology (Danvers, MA). Chemiluminescence from the samples was visualized using the Protein Simple Compass software.

### 2.14 Statistical Analysis

Data are expressed as mean ± standard error of the mean (SEM). SPSS 21.0 software (SPSS Inc.) was used to analyze the results. The significance of differences between groups was evaluated by non‐parametric one‐way analysis of variance (ANOVA) with the LSD post hoc test when no variance in homogeneity was found. Otherwise, the Mann–Whitney *U* non‐parametric test was performed. Significant differences were marked at *p < 0.05, **p < 0.01, ***p < 0.001 or #*p* < 0.05, ##*p* < 0.01.

## 3 Results

### 3.1 Molecular‐Weight Distribution and Monosaccharide Composition

**FIGURE 1:**
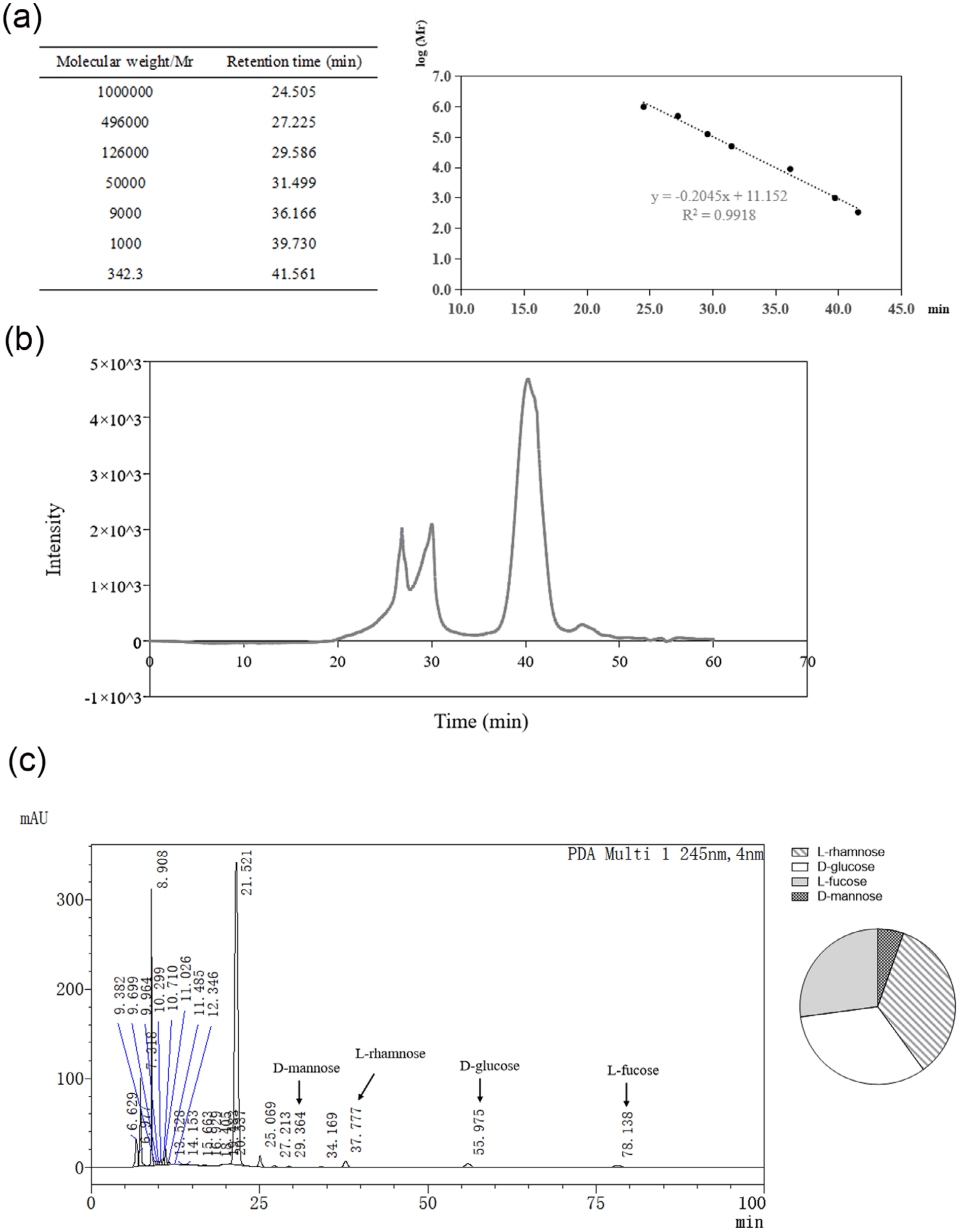
Molecular weight and distribution of PP. Molecular weight and distribution of PP. (a) The retention time (left) and calibration curve (right) of dextran standards. (b) HPGPC chromatogram of PP. (c) HPLC chromatogram of hydrolysate of PP.

Polysaccharides are one of the main active ingredients in *Polygonati Rhizoma* and are the quality standard (calculated by examining anhydrous glucose) of *Polygonati Rhizoma* (Chinese Pharmacopeia Commission 2020). Initially, the HPGPC method was used to detect the molecular weight of PP. The retention time and calibration curve of dextran standards are shown in Figure 1a. There are 3 main peaks on the HPGPC chromatogram (Figure 1b). Peak 1 and peak 2 represent large‐molecular‐weight polysaccharides (> 160 kDa), whereas peak 3 represents low‐molecular‐weight polysaccharides (~836 Da). Analysis of peak area (Table 1) reveals that the proportion of 836 kDa polysaccharides (Peak 3) in PP is 79.59%. Next, HPLC analysis shows that PP is mainly composed of L‐fucose, L‐rhamnose, D‐glucose, and D‐mannose, with a molar ratio of 1.00:1.28:1.21:0.20 (Figure 1c).

**TABLE 1:** Molecular weight and peak area of PP.

Peak number	Retention time (min)	Molecular weight	Ratio of peak area
1	26.361	592046.7	1
2	29.051	167338.5	1.21
3	40.331	836.5	8.62

### 3.2 Effect of PP on Uptake and Blood Glucose in *db/db* Mice

**FIGURE 2:**
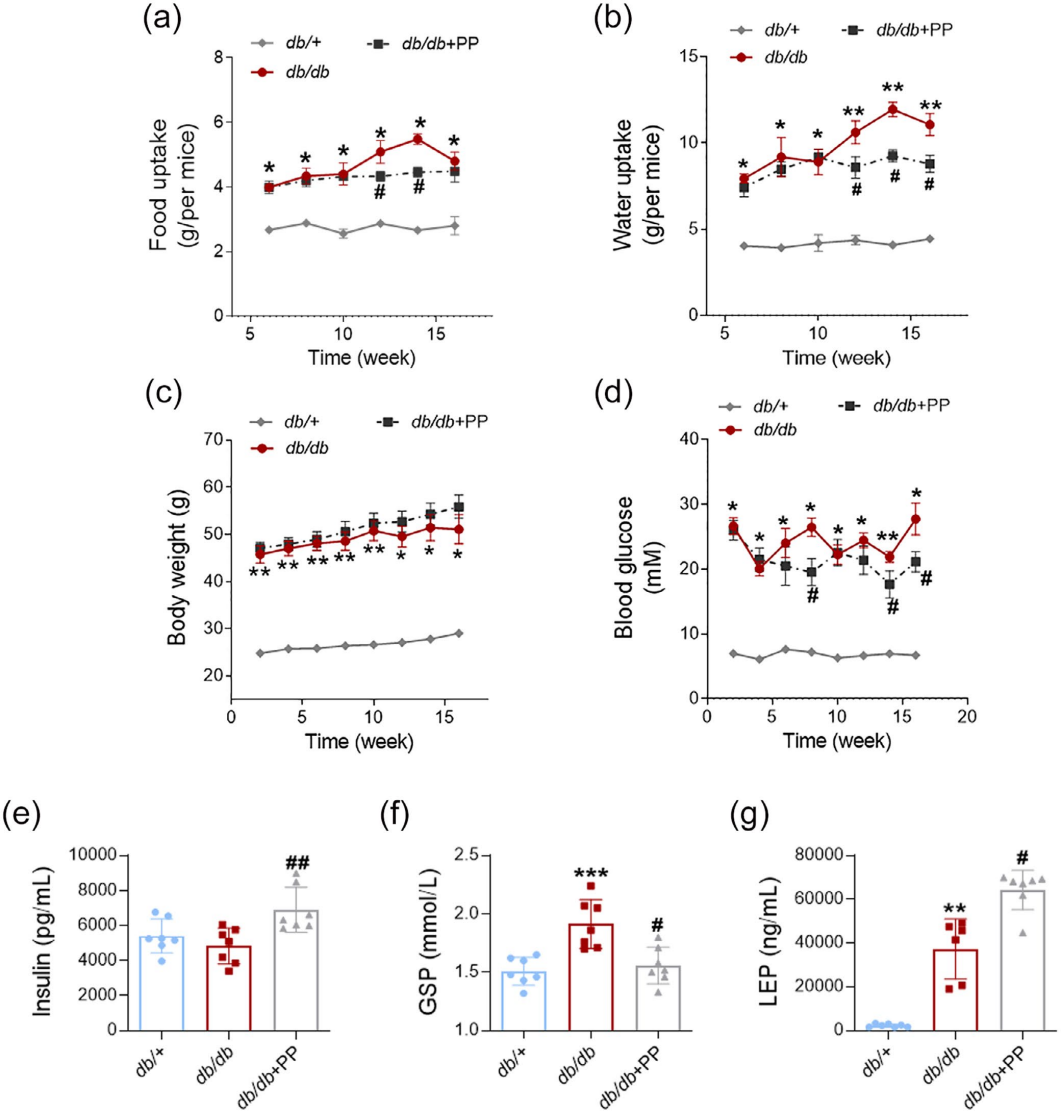
PP alleviated the blood glucose of *db/db* mice. PP alleviated the blood glucose of *db/db* mice. (a) Food uptake (*n* = 7). (b) Water uptake (*n* = 7). (c) Body weight (*n* = 7). (d) Blood glucose (*n* = 7). (e) Insulin level in serum (*n* = 7). (f) GSP level (*n* = 7). (g) LEP level in serum (*n* = 7). Data = Mean ± SEM. *p < 0.05, **p < 0.01, ***p < 0.001 versus *db/+*; #*p* < 0.05, ##*p* < 0.01 versus *db/db*.

Data in Figure 2a showed that PP (1.0 g/kg) slightly reduced the food uptake of *db/db* mice in the twelfth week and the fourteenth week. The water uptake of the PP‐treated group was also decreased in the last 6 weeks (Figure 2b). The body weight of *db/db* mice was higher than that of the NC group, and there was no alteration after PP (1.0 g/kg) treatment (Figure 2c). The next result showed that the blood glucose of *db/db* mice was significantly higher than that of *db/+* mice (Figure 2d). Meanwhile, the blood glucose levels of the PP + *db/db* group showed a decreasing trend from the eighth week, and the difference was significant in the eighth week, the fourteenth week, and the sixteenth week (Figure 2d). Administration of PP (1.0 g/kg) increased the content of serum insulin in *db/db* mice (Figure 2e). PP (1.0 g/kg) also restricted the elevation of GSP content in vivo (Figure 2f). Additionally, the serum LEP level in *db/db* mice was found to be increased, while PP (1.0 g/kg) further elevated the serum LEP content in *db/db* mice (Figure 2g). These results implied PP (1.0 g/kg) helped to moderate appetite and blood glucose in *db/db* mice.

### 3.3 PP Alleviated Kidney Dysfunction in *db/db* Mice

**FIGURE 3:**
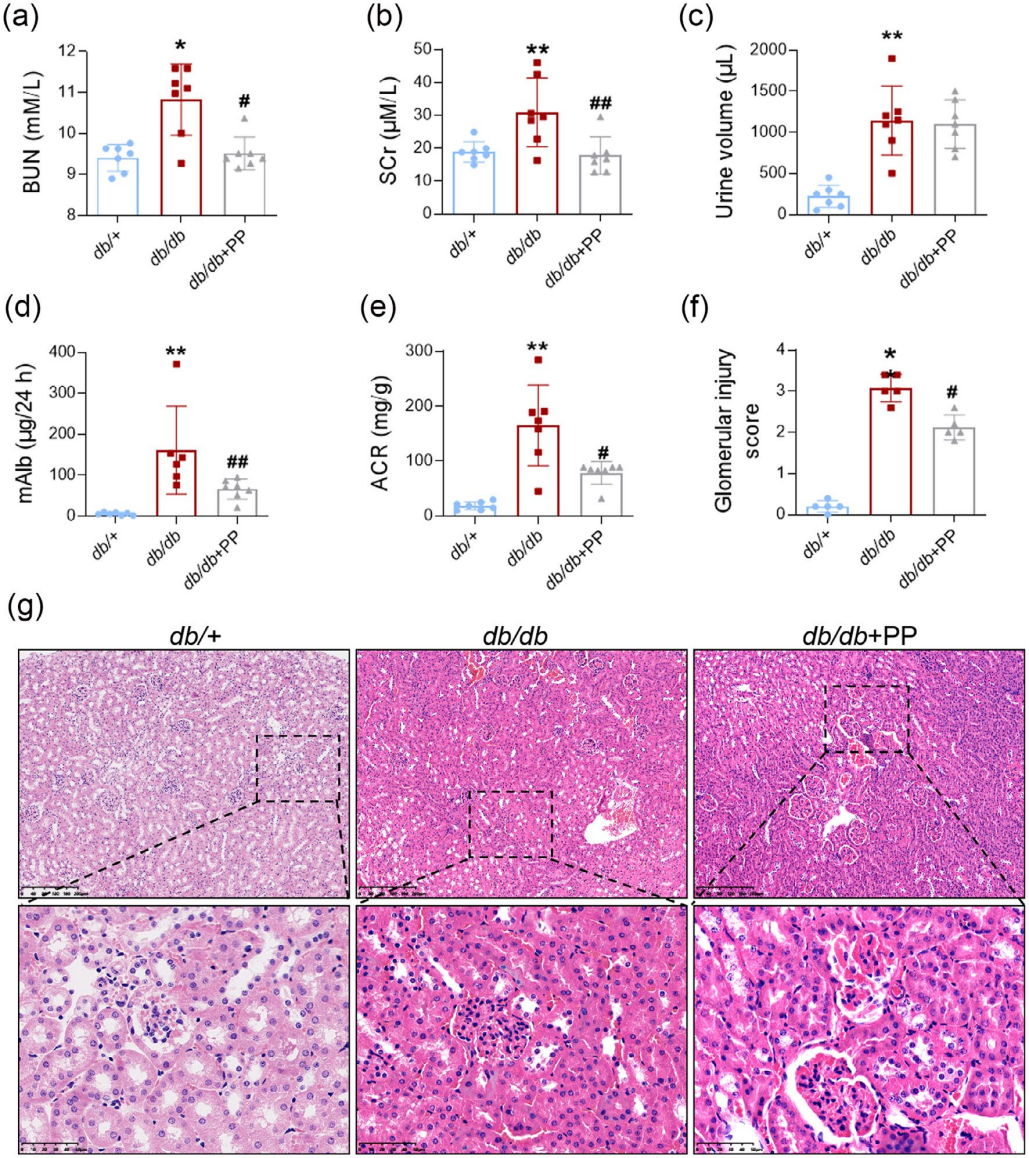
PP alleviated kidney dysfunction in *db/db* mice. PP ameliorated kidney dysfunction in *db/db* mice. (a) BUN level in serum (*n* = 7). (b) Creatinine level in serum (*n* = 7). (c) Urine volume (*n* = 7). (d) Urinary mAlb level (*n* = 7). (e) ACR level (*n* = 7). (f) Histopathologic scoring of glomerular injury (*n* = 5). (g) Representative image of H&E staining of kidneys from *db/db* or *db/+* mice. Data = Mean ± SEM. *p < 0.05, **p < 0.01 versus *db/+*; #*p* < 0.05, ##*p* < 0.01 versus *db/db*.

Renal function of animals was then assessed. BUN and Cr in serum were remarkably increased in the *db/db* group compared to the *db/+* mice, while PP reversed these (Figure 3a,b). Microalbuminuria is the earliest detectable clinical manifestation of DKD, and urine albumin‐creatinine ratio (ACR) reflects glomerular damage (Rossing and Epstein 2022). PP did not reverse the increased urine volume in *db/db* mice (Figure 3c). However, urinary mAlb and ACR value in *db/db* mice were much higher than that in *db/+* mice, while PP (1.0 g/kg) obviously alleviated these (Figure 3d,e). Next, the results of H&E and glomerular injury scores suggested that the kidneys in model mice showed glomerular deformation, hyperplasia of mesangial cells, and Bowman's space reduction, whereas PP markedly ameliorated these in *db/db* mice (Figure 3f,g). These results manifested that PP suppressed the progression of DKD in *db/db* mice.

### 3.4 PP Improved Lipid Homeostasis and Suppressed Oxidative Stress

**FIGURE 4:**
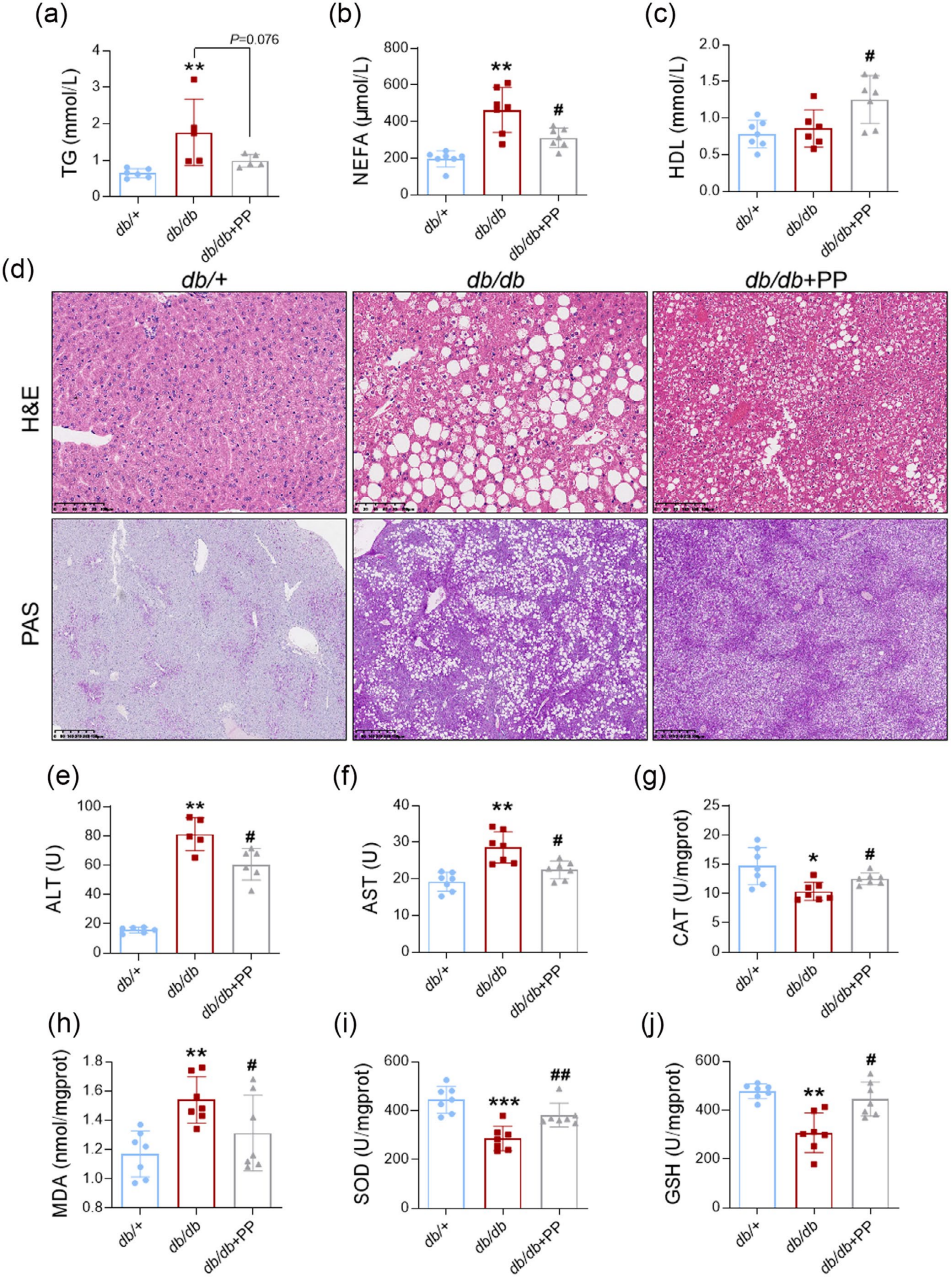
PP improved lipid metabolism and alleviated oxidative stress in *db/db* mice. PP improved lipid metabolism and alleviated oxidative stress in *db/db* mice. (a) TG level in serum (*n* = 5–6). (b) NEFA level in serum (*n* = 7). (c) HDL level in serum (*n* = 7). (d) Representative image of H&E staining or PAS‐staining of kidneys from *db/db* or *db/+* mice (*n* = 7). At least 3 fields were randomly selected and captured. (e) ALT level in serum (*n* = 6). (f) AST level in serum (*n* = 7). (g) CAT level in liver (*n* = 7). (h) MDA level in liver (*n* = 7). (i) SOD level in liver (*n* = 7). (j) GSH level in liver (*n* = 7). Data = Mean ± SEM. *p < 0.05, **p < 0.01, ***p < 0.001 versus *db/+*; #*p* < 0.05, ##*p* < 0.01 versus *db/db*.

Lipidtoxicity is one of the main causes of kidney injury in T2D (Opazo‐Ríos et al. 2020). The liver acts as the metabolic center of the body and is the main site for the production of lipid droplets and glycogen (Zheng et al. 2018). In this study, serum TG and NEFA content were increased in *db/db* mice, while PP (1.0 g/kg) significantly inhibited these (Figure 4a,b). PP (1.0 g/kg) also raised the HDL level in the serum of *db/db* mice (Figure 4c). Meanwhile, PP (1.0 g/kg) reduced hepatic steatosis and inflammation infiltration in *db/db* mice (Figure 4d). Hepatic glycogen was visibly accumulated in *db/db* mice, while PP (1.0 g/kg) had no effect on this (Figure 4d). Besides, PP (1.0 g/kg) slightly reduced the rise of ALT and AST levels in *db/db* mice (Figure 4e,f). The levels of CAT, SOD, and GSH‐Px were increased in *db/db* mice, while PP (1.0 g/kg) reversed all these phenomena individually (Figure 4g–j). PP also increased the diminished level of MDA in *db/db* mice. This information suggested PP (1.0 g/kg) improved lipid homeostasis and inhibited oxidative stress in *db/db* mice. Moreover, PP (10, 30 μg/mL) alleviated the elevated lipid accumulation and ROS levels in 0.5 mM NEFA‐induced AML‐12 cells (Figure S2). However, H_2_O_2_ (100 μM) could not totally block the PP‐provided amelioration of lipid accumulation in 0.5 mM NEFA‐induced AML‐12 cells (Figure S2). It seems that anti‐oxidative stress may not be the most critical mechanism involved in the benefit of PP on lipid metabolism.

### 3.5 PP Attenuated Renal Fibrosis in *db/db* Mice

Low‐grade inflammation is commonly detected in the early stage of DKD (Jung and Yoo 2022). Q‐PCR results implied that PP (1.0 g/kg) could reverse the increased renal mRNA level of *Il6* (gene of IL‐6) and *CCL2* (gene of MCP‐1) in *db/db* mice (Figure S1). PP (1.0 g/kg) visibly reversed the IL‐6 content in renal tissues from *db/db* mice, whereas the reduction of TNF*ɑ* expression in the PP‐treated group was limited (*p* > 0.05) (Figure S3).

**FIGURE 5:**
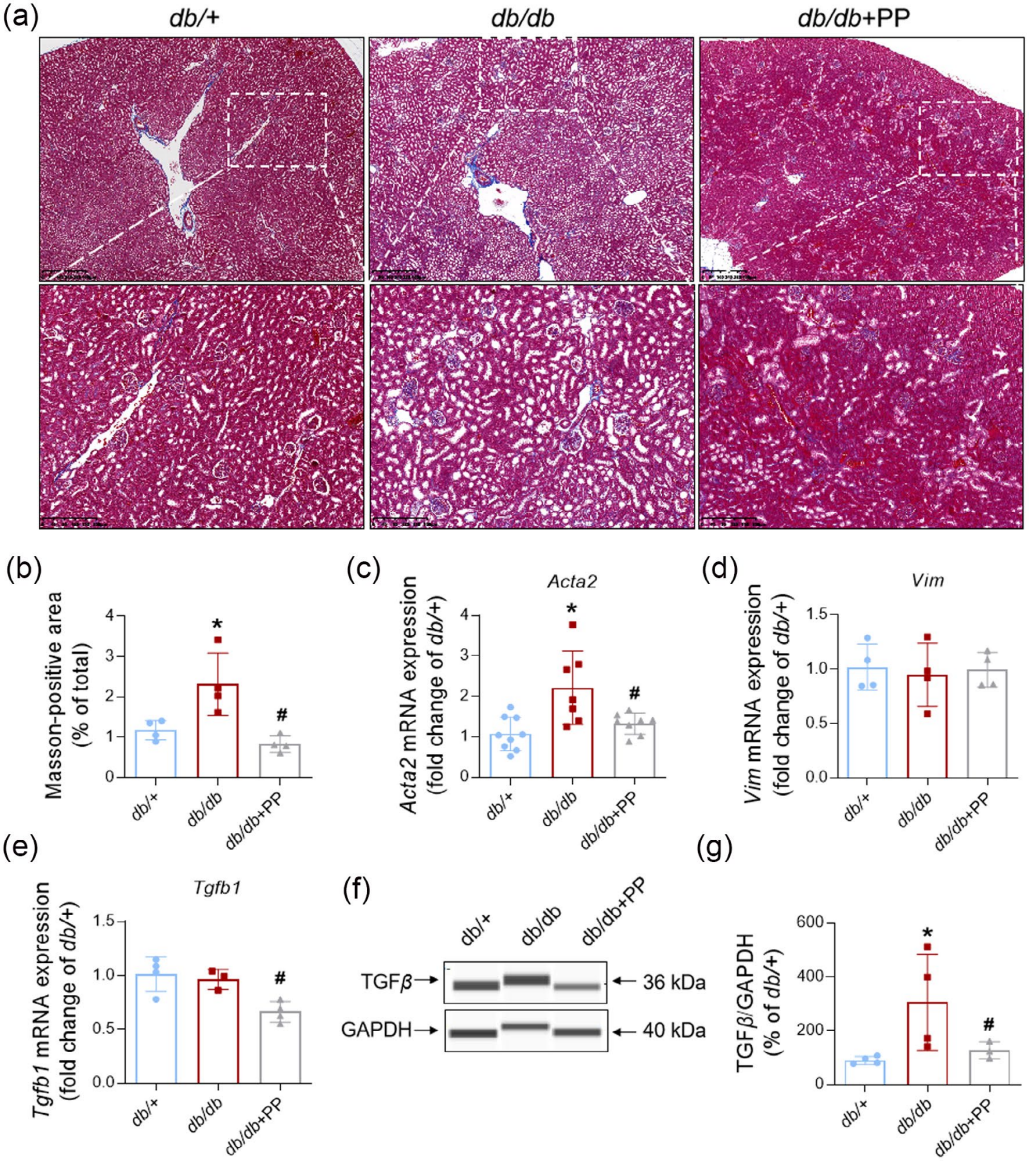
PP ameliorated renal fibrosis in kidneys from *db/db* mice. (a, b) Representative image (upper, scale bar: 400 μm) (lower, scale bar: 200 μm) and quantification of Masson‐staining of kidneys from *db/db* or *db/+* mice (*n* = 7). (c–e) Renal mRNA expression of *Acta2* (c, *n* = 7), *Vim* (d, *n* = 4), *Tgfb1* (e, *n* = 4). (f, g) Representative image and quantification of TGF*β* expression in kidneys from *db/db* or *db/+* mice (*n* = 4). Data = Mean ± SEM. *p < 0.05 versus *db/+*; #*p* < 0.05 versus *db/db*.

Fibrosis is one of the most important characteristics in the later stage of DKD (Thomas et al. 2015). Results of Masson‐staining demonstrated that tissue fibrosis appeared in kidneys from *db/db* mice, whereas the Masson‐positive area was remarkably diminished in the *db/db* + PP group (Figure 5a,b). Meanwhile, PP (1.0 g/kg) significantly reduced the upregulated expression of *Acta2* in renal tissues from *db/db* mice (Figure 5c), while no significant difference was found in the renal mRNA level of *Vim* among the three groups (Figure 5d). PP (1.0 g/kg) downregulated the mRNA level of *Tgfb1* in kidneys from *db/db* mice (Figure 5e). PP (1.0 g/kg) also significantly suppressed the elevated content of TGF*β* in vivo (Figure 5f,g). Expression of inflammatory and fibrosis‐related genes in GECs isolated from mice showed a similar trend in the PP‐treated group (Figure S4). These results suggested that PP ameliorated renal fibrosis and expression of TGF*β* in *db/db* mice.

### 3.6 PP Promoted the Expression of Smad7 and Blocked the Phosphorylation of Smad2

**FIGURE 6:**
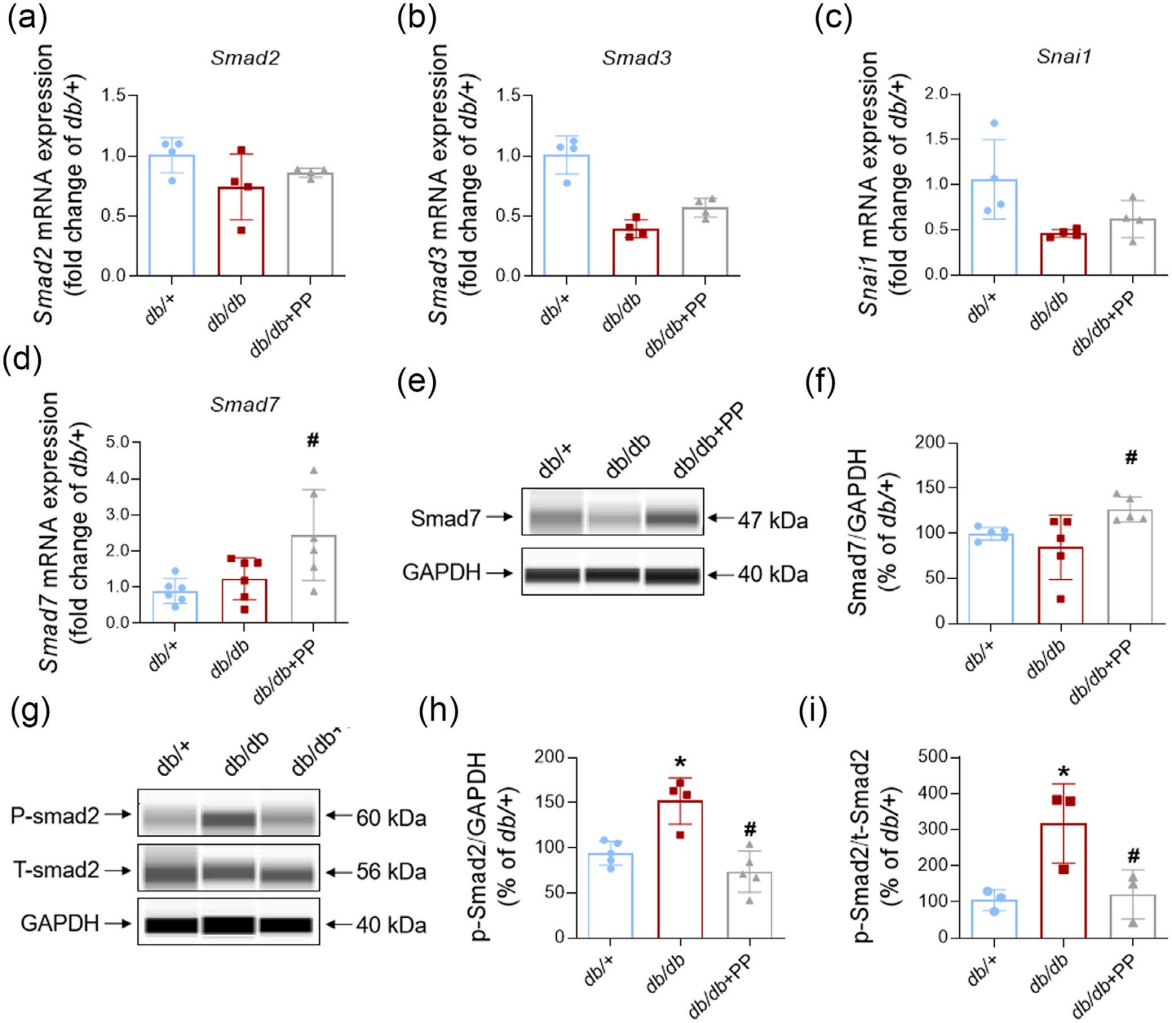
PP promoted the expression of Smad7 and blocked the phosphorylation of Smad2. PP promoted the expression of Smad7 and blocked the phosphorylation of Smad2. (a–d) Renal mRNA expression of Smad2 (*n* = 4), Smad3 (*n* = 4), Snail1 (*n* = 4), and Smad7 (*n* = 6). (e, f) Representative image (e) and quantification (f) of Smad7 expression in kidneys from *db/db* or *db/+* mice (*n* = 5). (g–i) Representative image (g) and quantification of Smad2 phosphorylation (h, i) in kidneys from *db/db* or *db/+* mice (*n* = 3). Data = mean ± SEM. *p < 0.05 versus *db/+*; #*p* < 0.05 versus *db/db*.

TGF*β* promotes renal fibrosis mainly through Smad and Snail signals (Frangogiannis 2020). Smad2/3 is critically involved in the activation of TGF*β*/Smad signal pathway, while Smad7 always acts as an inhibitor of Smad2/3 (Frangogiannis 2020). Data in Figure 6a–c manifested that the renal mRNA expression of *Smad2*, *Smad3*, and *Snail1* had no visible change. However, the renal mRNA of *Smad7* in the PP‐treated group had a larger quantity than in the *db/db* group (Figure 6d). Results of simple Western suggested that PP remarkably enhanced the renal protein content of Smad7 in vivo (Figure 6e,f). Consistently, the phosphorylation of Smad2 was significantly increased in kidneys from *db/db* mice, while PP successfully reduced this (Figure 6g–i).

## 4 Discussion

As the (KDIGO 2022) Clinical Practice Guideline prompted, people with DM and DKD should get more intricate nutrient intake (Navaneethan et al. 2023). A regular consumption of functional foods has great physiological benefits for controlling T2D, as well as its complications (Alkhatib et al. 2017). *Polygonati Rhizoma* has been included in China's Medicinal Food Directory (Gong et al. 2023). Polysaccharides, consisting of high‐molecular‐weight carbohydrates, are the main active ingredients of *Polygonati Rhizoma* (Chinese Pharmacopeia Commission 2020; Gong et al. 2023). Recently, PP got much attention for its effect on preventing and attenuating diabetes (Liu et al. 2022). Clinical feedback has demonstrated that the quality of carbohydrates is essential for managing DM (Sluijs et al. 2010; AlEssa et al. 2015). A previous study showed that the aqueous extract of *Polygonati Rhizoma* could ameliorate glucose metabolism in streptozotocin‐induced diabetic mice (Wang, Liu, et al. 2022). We have previously reported that PP slightly moderated the serum glucose in 15‐week‐old *db/db* mice with early‐stage T2D (Chen et al. 2023). However, the role of PP in the treatment of DKD was unclear.

First of all, the distribution of polysaccharides in PP was determined by using the HPGPC method. There were three main peaks on the HPGPC chromatogram. Low‐molecular‐weight polysaccharides (~836 Da) account for the highest proportion in PP. HPLC analysis revealed that the hydrolysate of PP included rhamnose, glucose, fucose, and mannose. Results of the animal experiment showed that the administration of PP repressed the uptake of food and water in *db/db* mice in the last 6 weeks. The blood glucose level in the PP‐treated group showed a decreasing trend from the sixth week of the experiment, which was earlier than the loss of appetite in *db/db* mice. GSP reflects the average level of blood glucose over 1–2 weeks. PP reduced the elevated GSP content in *db/db* mice. These results implied a hypoglycemic effect beyond the uptake inhibition. Our results showed that the serum insulin level of *db/db* mice was increased in the PP‐treated group. It seems that the elevation of serum LEP level in *db/db* mice is a compensatory response to the lack of LEP receptor, while it is worth noting that PP further upregulated the LEP content in vivo. A previous study has reported that LEP acted upstream in the regulation of insulin secretion (Cong et al. 2007). These results suggested that PP might help to ameliorate glycometabolism in *db/db* mice.

DKD is a devastating microvascular complication associated with DM. Microalbuminuria, a sensitive marker for glomerular hyperfiltration, is the early manifestation of DKD (A/L et al. 2019). Lacking early intervention, approximately 50% of patients with microalbuminuria will progress to macroalbuminuria, highly increasing the risk of ESRD (Papadopoulou‐Marketou et al. 2017). Microalbuminuria occurs as a result of this endothelial dysfunction (A/L et al. 2019). A previous study has shown that PP could attenuate diabetic microvascular complication retinal injury in a diabetic rat model (Wang et al. 2019). A Chinese herbal complex that contains *Polygonati Rhizoma* could ameliorate nephrotoxicity induced by cisplatin in mice (Guo et al. 2018). Recently, PP treatment was found to constrict both SCr and BUN levels in potassium oxonate and hypoxanthine‐induced hyperuricemia in vivo (Zhang et al. 2024). However, the effect of PP on DKD was still unknown. In this study, the elevated BUN and serum creatinine (SCr) values indicated kidney injury in the *db/db* group, while PP successfully rescued these. Next, PP also significantly inhibited the increase of mAlb and ACR levels in *db/db* mice. It seems that PP might ameliorate the permeability of glomerular endothelium in *db/db* mice. PP also mitigated the glomerular injury scores and the morphological changes of DKD, including glomerular deformation, Bowman's space reduction, and interstitial inflammation infiltration in vivo. These data manifested PP inhibited the progression of DKD in *db/db* mice.

DKD is initiated by diabetes‐related disturbances in glucose and lipid metabolism (Jung and Yoo 2022). Notably, the blood glucose level in the PP‐treated group remained approximately 20 mM, which was still far outside the range in the normal group. It suggested additional insults might play a critical role as well. Dyslipidemia is a critical stimulation for inflammation, oxidative stress, and apoptosis in DKD (Opazo‐Ríos et al. 2020). In this study, PP suppressed the large production of TG and NEFA and encouraged the elevation of serum HDL content in *db/db* mice. T2D is an important risk factor of metabolic‐associated fatty liver disease (MAFLD), which is characterized by fatty liver, hepatocyte injury, dyslipidemia, liver inflammation, and oxidative stress (Ullah et al. 2019). In this study, PP attenuated hepatic steatosis in 26‐week‐old *db/db* mice, while the hepatic glycogen had no obvious alteration. It seems that PP was more effective in lipid metabolism. Oxidative stress and lipotoxicity promote the progression of T2D and MAFLD and are now increasingly recognized in DKD pathogenesis (Demir et al. 2021; Schelling 2022). CAT, SOD, and GSH serve as abundant and important antioxidants in this situation (Catherwood et al. 2002; Li et al. 2021). A previous study reported that an aqueous extract of *Polygonati Rhizoma* (dosage at 5 g/kg) could ameliorate liver injury in ethanol‐induced mice (Wang et al. 2021). Our result showed that PP reduced ALT and AST levels in *db/db* mice. Meanwhile, PP significantly rescued the alteration of CAT, SOD, MDA, and GSH in *db/db* mice. These results imply that PP improved lipid metabolism and alleviated oxidative stress in *db/db* mice. Hyperlipidemia is a major cause of oxidative stress in T2D, while oxidative stress also conversely accelerates the disorder of lipid metabolism (Sottero et al. 2015; An et al. 2023). Our results showed that PP attenuated the lipid accumulation and the increase of ROS in NEFA‐induced AML‐12 cells, while H_2_O_2_ did not totally block the diminishment of lipid droplet formation in the PP + NEFA group. Interestingly, previous studies have found the direct hypolipidemic effect of natural fucoidan fractions and derivative polysaccharide of rhamnose in vivo and in vitro (Wang, Lu, et al. 2022; Ren et al. 2019), while L‐fucose and L‐rhamnose account for 61.7% of PP hydrolysates in our data. These evidences imply that PP has both direct hypolipidemic effects and mild antioxidant activity, which might further improve the interaction between dyslipidemia and oxidative stress.

Metabolites‐triggered inflammatory and fibrotic processes heavily contribute to the pathogenesis and progression of DKD (Thomas et al. 2015; Tang and Yiu 2020). Low‐grade inflammation, but not severe inflammation, is typical in the development of DKD (Jung and Yoo 2022). PP has been reported to have a good anti‐inflammatory effect in acute lung injury and Alzheimer's disease in vivo (Liu et al. 2020; Luo et al. 2022). Our results showed PP obviously inhibited the increased expression of *Il6* and *Ccl2* in *db/db* mice, while the decrease of TNF*α* expression in the PP‐treated group was not sufficient. It seems that the effect of PP on renal inflammation in *db/db* mice was limited. Long‐term chronic inflammation accompanied by diabetic metabolites will promote the development of renal fibrosis in DKD (Thomas et al. 2015; Tuleta and Frangogiannis 2021). However, few studies explore the effect of PP on fibrosis. In our study, PP suppressed the elevated Masson‐positive area in the renal section of *db/db* mice. PP also reduced the expression of *Acta2* (known as *ɑ‐*SMA) and *Tgfb1* (known as TGF*β*) in kidneys from *db/db* mice. Our results suggest that PP could reduce renal fibrosis in *db/db* mice.

TGF*β*/Smad and TGF*β*/Snail signaling pathways are two classical regulators of fibrosis (Tuleta and Frangogiannis 2021). Smad2/3 is the most important nuclear transcription factor in the TGF*β*/Smad signaling pathway, while Smad7 is a negative regulator of TGF*β‐Smad2/3* signal pathway (Tuleta and Frangogiannis 2021; Lee and Massagué 2022; Hu et al. 2021). Polysaccharides from natural plants such as Dendrobium officinale, Astragalus, and phellinus igniarius have been reported to alleviate organic fibrosis by regulating TGFβ‐Smad signaling pathway (Wang, Ye, et al. 2022). Our results revealed that the transcription of *Smad2*, *Smad3*, and *Snai1* had no alteration after PP administration, while the PP‐treated *db/db* mice displayed a high level of Smad7 mRNA. PP also significantly increased the protein expression of Smad7 in kidneys from *db/db* mice. Phosphorylation of Smad2/3 is required for its accumulation in the cellular nucleus (Tuleta and Frangogiannis 2021). Smad7 provides competition for TGF*β* type‐1 receptor (TGF*β*RI) and then blocks the phosphorylation of Smad2/3 (de Ceuninck et al. 2020). In this study, PP abrogated the increased phosphorylation of Smad2 in the kidneys of *db/db* mice. A previous study about relieving fibrosis in DKD focused on how to directly block TGF*β* and downstream phosphorylation of Smad2/3 (Tuleta and Frangogiannis 2021; Jiang et al. 2021). Thus, it can be seen that there is a lack of safe and effective treatment targeting Smad7 in DKD. Our results revealed that PP ameliorated renal fibrosis by upregulating the expression of Smad7 and inhibiting the activation of TGF*β‐Smad2/3* signal pathway in kidneys from *db/db* mice. Additionally, Smad7 has been reported to alleviate inflammation in autoimmune and inflammatory diseases (Lin et al. 2017; He et al. 2023). This indicates the potential involvement of Smad7 in PP‐provided amelioration of low‐grade inflammation in DKD, which needs to be further investigated.

This study has several limitations. First, we explored the protective effect of PP against DKD, but did not fully compare the beneficial effects of other components in *Polygonati Rhizoma*, such as saponins, flavonoids, or alkaloids. Second, only T2D *db/db* mice were investigated; therefore, our results may not apply to T1D conditions. Third, the major bioactive component in PP could be further identified; the result of HPGPC suggested the need for separation of homogeneous polysaccharids to validate our findings.

Despite the limitations, this study is the first to provide insight into the potential effect and mechanism of natural product PP on T2D‐triggered DKD. Moreover, we previously found that PP could reduce the intestinal abundance of the Lachnospiraceae family and the Romboutsia genus microbes in *db/db* mice (Chen et al. 2023). These genera were found to be positively correlated with hyperglycemia and hyperlipidemia, affecting the production of propionic acid (Li, Xu, et al. 2020; Wang et al. 2020). Therefore, the alteration of metabolic products involved in our study warrants further analysis and validation to clarify the underlying mechanisms.

## 5 Conclusion

**FIGURE 7:**
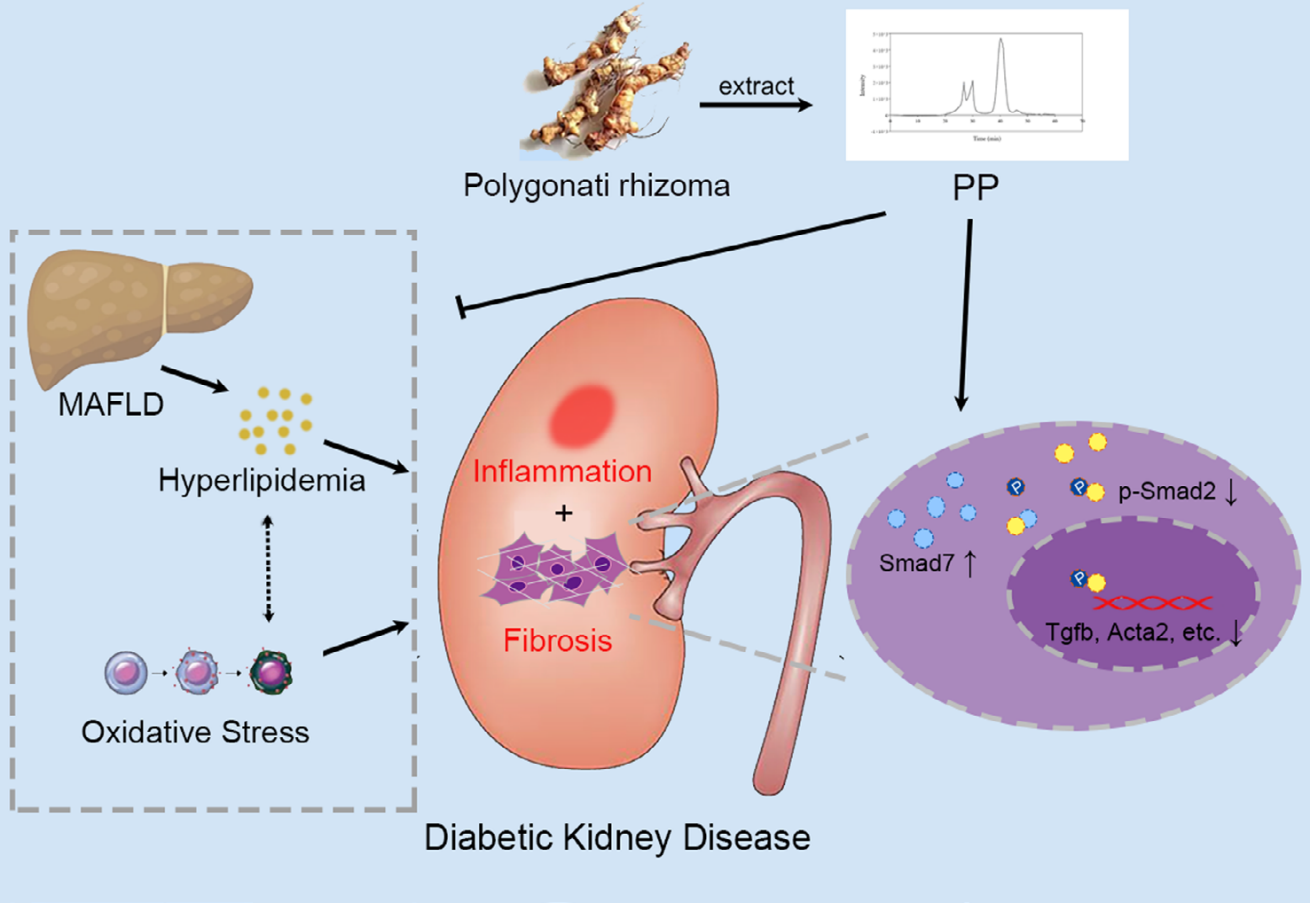
A schematic presentation of the effect of PP on DKD in T2D.

In conclusion, we demonstrated that PP attenuated renal dysfunction and fibrosis in *db/db* mice through ameliorating lipid metabolism, anti‐oxidative stress, and inhibiting the activation of TGF*β‐Smad2* signaling pathway (Figure 7). This study reveals that natural extraction of PP might be a promising functional food for preventing and treating DKD. This study also provides insights into the mechanisms by which PP alleviates DKD.

## Author Contributions

**Xiyu Mei:** conceptualization (lead), data curation (lead), formal analysis (lead), investigation (equal), methodology (lead), resources (equal), software (equal), validation (equal), visualization (equal), writing – original draft (lead), writing – review and editing (equal). **Zeming Ren:** data curation (equal), funding acquisition (supporting), investigation (equal), resources (equal), software (equal), validation (equal). **Ziyun Gao:** data curation (equal), formal analysis (equal), investigation (equal), validation (equal), visualization (equal). **Sisi Chen:** data curation (equal), formal analysis (equal), investigation (equal). **Xuan Chen:** funding acquisition (supporting), investigation (equal), methodology (supporting), supervision (equal). **Qingyun Zhou:** data curation (equal), formal analysis (supporting), investigation (supporting). **Yeling Tong:** methodology (supporting), supervision (equal), validation (supporting). **Guanhai Dai:** conceptualization (equal), funding acquisition (lead), methodology (equal), project administration (lead), resources (lead), writing – review and editing (equal).

## Conflicts of Interest

The authors declare no conflicts of interest.

## Supporting information


Figures S1–S4.

Table S1.

## Data Availability

The data presented in this study are available on request from the corresponding author.
