# Supplementary figures and images for: Polygonati Rhizoma Polysaccharides Ameliorated Diabetic Kidney Disease in db/db Mice via Inhibiting TGFβ /Smad2 Signaling Pathway
Source: Food Sci Nutr. 2025 Jul 18;13(7):e70677. doi: 10.1002/fsn3.70677 (PMC12274161; doi:10.1002/fsn3.70677)

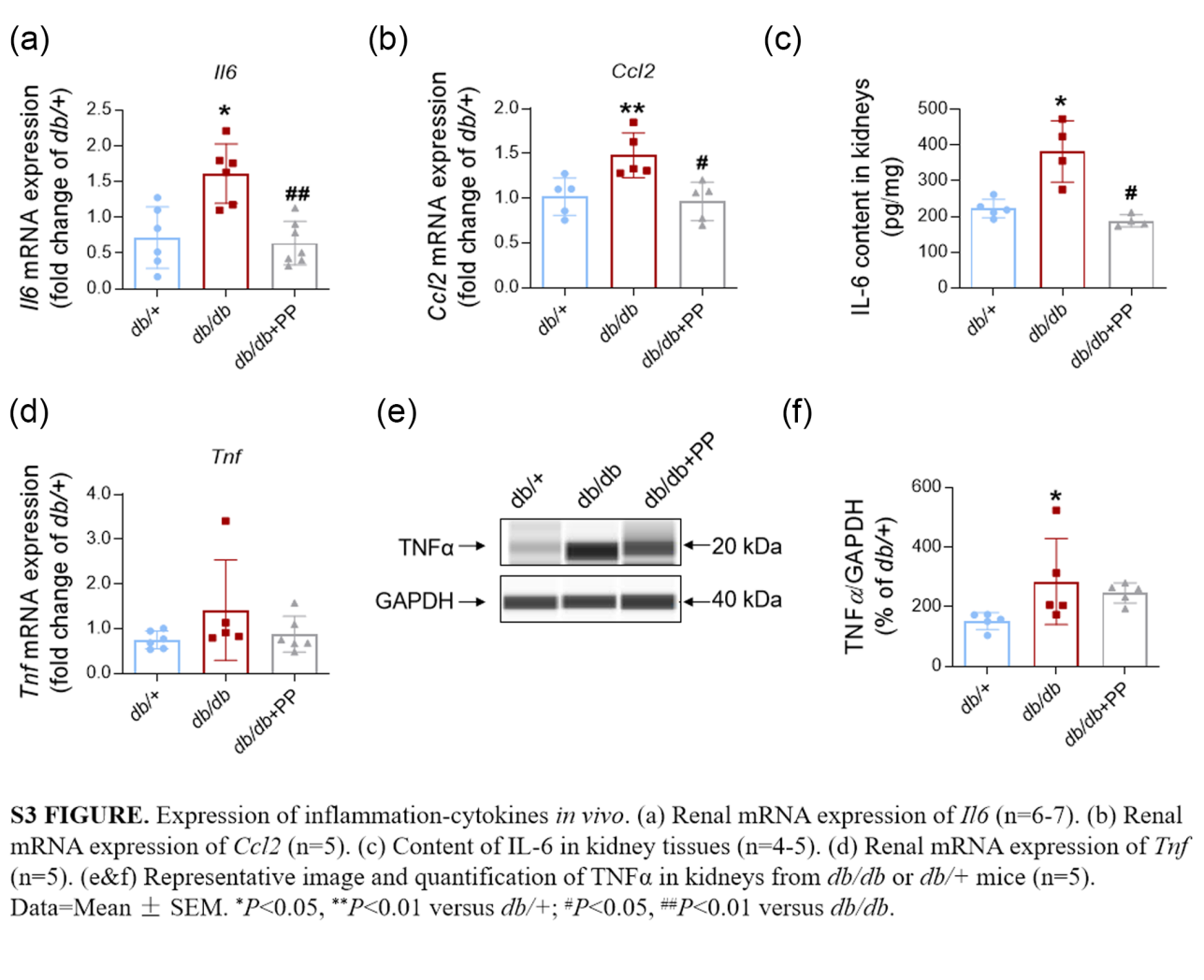


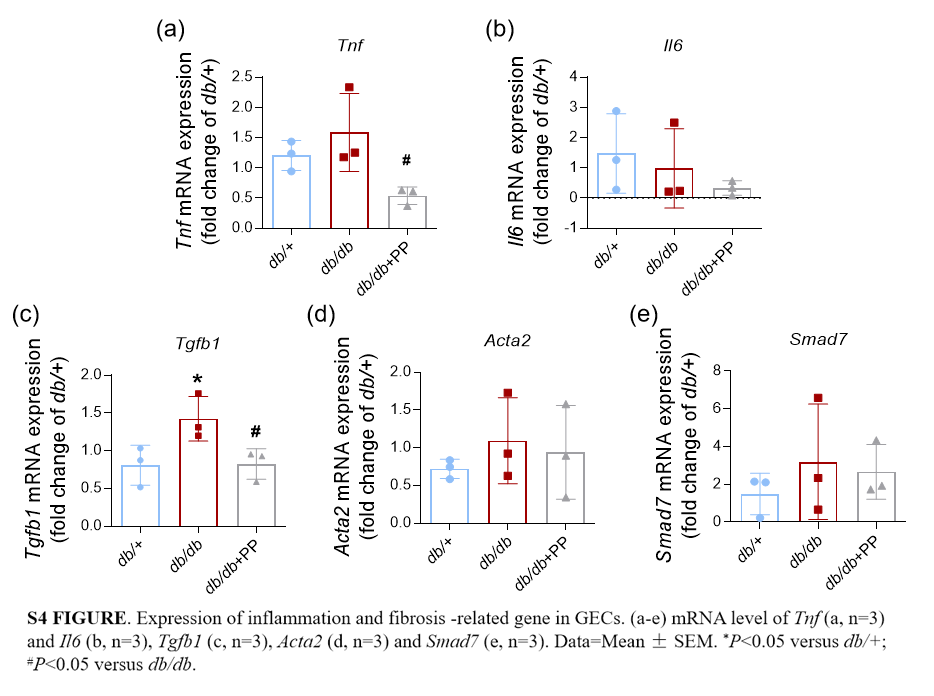

Supplement: Supplementary file 1 — Figures S1–S4. [file FSN3-13-e70677-s002.docx]
