# Supplementary material for: Polygonati Rhizoma Polysaccharides Ameliorated Diabetic Kidney Disease in db/db Mice via Inhibiting TGFβ /Smad2 Signaling Pathway
Source: Food Sci Nutr. 2025 Jul 18;13(7):e70677. doi: 10.1002/fsn3.70677 (PMC12274161; doi:10.1002/fsn3.70677)
Supplement: Supplementary file 2 — Table S1. [file FSN3-13-e70677-s001.docx]

**Supplementary Table 1.** List of Primers for Real-time PCR

| Target | Gene ID | Protein | Primer | Sequence |
| --- | --- | --- | --- | --- |
| *Actb* | 11461 | β-Actin | FP | 5'-TTCGTTGCCGGTCCACACCC-3' |
|  |  |  | RP | 5'-GCTTTGCACATGCCGGAGCC-3' |
| *Il6* | 16193 | IL-6 | FP | 5'-CGGAGAGGAGACTTCACAGAG-3' |
|  |  |  | RP | 5'-ATTTCCACGATTTCCCAGAG-3' |
| *Ccl2* | 20296 | MCP-1 | FP | 5'-CCACTCACCTGCTGCTACTCATTC-3' |
|  |  |  | RP | 5'-CTTCTTTGGGACACCTGCTGCTG-3' |
| *Tnf* | 21926 | TNF-ɑ | FP | 5'-AGGCACTCCCCCAAAAGAT-3' |
|  |  |  | RP | 5'-CAGTAGACAGAAGAGCGTGGTG-3' |
| *Tgfb1* | 21803 | TGF-β | FP | 5'-GTGTGGAGCAACATGTGGAACTCTA-3' |
|  |  |  | RP | 5'-TTGGTTCAGCCACTGCCGTA-3' |
| *Snai1* | 20613 | Snail | FP | 5'-TTCACCTTCCAGCAGCCCTAC-3' |
|  |  |  | RP | 5'-CTTTTGCCACTGTCCTCATCG-3' |
| *Smad2* | 17126 | Smad2 | FP | 5'-GTCGTCCATCTTGCCATTCACTC-3' |
|  |  |  | RP | 5'-TCTCCACCACCTGCTCCTCCAG-3' |
| *Smad3* | 17127 | Smad3 | FP | 5'-CCCACCACCCAGCAGTACAAC-3' |
|  |  |  | RP | 5'-AGCCACAGCCGTCCTCATCC-3' |
| *Smad7* | 17131 | Smad7 | FP | 5'-AAGGCAGTGACCACAAGGAATGAG-3' |
|  |  |  | RP | 5'-CACCCAGGCAACTTGACCCATAG-3' |
| *Vim* | 22352 | Vimentin | FP | 5'-CGTGAATACCAAGACCTGCTC-3' |
|  |  |  | RP | 5'-GGAAAAGTTTGGAAGAGGCAG-3' |
| *Acta2* | 11475 | ɑ-SMA | FP | 5'-GGGAGTAATGGTTGGAATGG-3' |
|  |  |  | RP | 5'-GGTGATGATGCCGTGTTCTA-3' |

FP, Forward Primer; RP, Reverse Primer
